# Factors influencing withdrawal of life-sustaining treatments in patients with severe acquired brain injuries: a scoping review

**DOI:** 10.3389/fneur.2026.1755086

**Published:** 2026-03-04

**Authors:** Alexia Abboud, Rose Jutras, Catherine Rollin, Loretta Norton, Stefanie Blain-Moraes, Catherine Duclos

**Affiliations:** 1 Department of Medicine, Université de Montréal, Montréal, QC, Canada; 2 Hôpital du Sacré-Cœur de Montréal, Centre Intégré Universitaire de Santé et de Services Sociaux du Nord-de-l'Île-de-Montréal, Montréal, QC, Canada; 3 Department of Neuroscience, Université de Montréal, Montréal, QC, Canada; 4 Nursing Sciences Faculty, Université de Montréal, Montréal, QC, Canada; 5 Department of Psychology, King’s University College at Western University, London, ON, Canada; 6 Western Institute for Neuroscience, Western University, London, ON, Canada; 7 Atlantic Fellows Program, Global Brain Health Institute, Trinity College, Dublin, Ireland; 8 Research Institute of the McGill University Health Center, Montréal, QC, Canada; 9 School of Physical and Occupational Therapy, McGill University, Montréal, QC, Canada; 10 Department of Anesthesiology and Pain Medicine, Université de Montréal, Montréal, QC, Canada

**Keywords:** anoxic brain injury, brain injuries, end of life care decision, intensive care units, stroke, surrogate decision maker (making), TBI - traumatic brain injury, withdrawal of life sustaining therapies

## Abstract

**Background:**

Withdrawal of life-sustaining treatments (WLST) is a leading cause of death in patients with severe acquired brain injuries (ABI). These decisions often occur under conditions of prognostic uncertainty and time-critical therapeutic windows and may be shaped by a complex interplay of factors. Elucidating these influences is essential to ensure that WLST decisions are made in an informed, unbiased, and transparent manner, and in alignment with wishes of the patients as well as their surrogate decision makers.

**Objective:**

Conduct a scoping review of literature to identify, elaborate and analyze the various factors that influence decisions to WLST in adult patients with ABI. This review aims to provide a comprehensive understanding of current practices.

**Methods:**

This scoping review, conducted according to PRISMA-ScR guidelines, examined literature on WLST in adult ABI, in whom brain death had not been declared. The search was conducted in PubMed and Web of Science, up to August 2024. Studies were screened by title/abstract and full text, with data systematically extracted. Only original, peer-reviewed articles focusing on WLST in adult severe ABI patients were included. *N* = 2,963 independent papers were initially found, of which *N* = 2,881 were excluded. A final count of *N* = 81 independent papers were included.

**Results:**

Demographic factors (age, sex, race, socioeconomic status, etc.; *n* = 50), prognosis and clinical factors (*n* = 59), family preferences (*n* = 28), physician-related factors and institutional context (*n* = 31), formal medical directive (*n* = 13), ethical/legal frameworks (*n* = 13), geographical differences (*n* = 9) and religious beliefs (*n* = 5) all played pivotal roles in WLST decisions. Older age consistently emerged as a determinant for WLST, as well as poor prognosis and white race.

**Conclusion:**

WLST decisions are most often made for older adults, with age consistently identified as a key predictor, independent of the clinical severity of ABI. Additional factors such as race, socioeconomic status, advance directives, and variations in healthcare provider attitudes and institutional policies further contribute to disparities in WLST practices. Understanding these intersecting influences is essential to recognizing potential biases and promoting more equitable, patient-centered end-of-life decision-making.

## 1 Introduction

Advances in medical technology have made it possible to sustain life in situations that were once considered unsurvivable, shifting ethical and clinical questions from “Can we keep this patient alive?” to “Should we?” It has therefore become the clinician’s role to both provide and, when appropriate, withdraw life sustaining treatments (WLST). The latter can be defined as “the cessation and removal of an ongoing medical therapy with the explicit intent not to substitute an equivalent alternative treatment. It is fully anticipated that the patient will die following the change in therapy” (1). Such measures usually include interruption of mechanical ventilation, vasopressors, artificial nutrition, and hydration. Withdrawing these treatments represents a transition from curative to palliative intent.

This decision-making process is highly influenced by the principle of self-determination, which grants patients the right to accept or refuse any medical intervention (2, 3). However, when patients with severe acquired brain injuries (ABI) lose the capacity to make or communicate decisions, responsibility shifts to a surrogate decision-maker, typically a close family member or a legal representative, who must make these choices in collaboration with the clinical team.

The conversations leading up to, and the act of WLST, are shaped by multiple, intersecting factors, including medical, legal, ethical, cultural, religious, and family-related considerations. WLST is a particularly sensitive process, as healthcare providers must often navigate without a clear prognosis, while families cope with grief and ambiguity. Those decisions are therefore made on a case-by-case basis.

This contextual approach gives rise to two major challenges: (1) it places the burden of decision-making on families and clinicians, who may feel distressed over their perceived role in the death of a patient or loved one (4, 5); (2) the lack of structured recommendations makes the process vulnerable to subjective judgments and potential biases (6).

While standardized guidelines may not capture the individualized nature of end-of-life decisions, a better understanding of how such decisions are currently made can help reassure families and clinicians that their patient-centered choices are neither isolated nor arbitrary. It can also prompt critical reflection on the potential biases that influence the decision-making process. Although WLST has been extensively studied in contexts like terminal cancer, and the prevalence of WLST has been broadly examined, research specifically focused on how these decisions are made for unresponsive patients with severe ABI is relatively scarce. To our knowledge, no comprehensive synthesis of this literature currently exists, hindering the development of evidence-based approaches to patient care. The aim of this review is to map existing literature and identify key factors that influence WLST decision in severe ABI patients, specifically in intensive care units (ICU).

## 2 Methods

### 2.1 Search strategy

A comprehensive scoping review was conducted in accordance with PRISMA-ScR guidelines (7). An initial exploratory search of PubMed and Web of Science was conducted to identify relevant keywords and index terms appearing in titles and abstracts, which were subsequently applied systematically across the selected databases. All identified keywords and controlled vocabulary (e.g., MeSH terms and subheadings) were adapted to each database’s indexing system to ensure comprehensive coverage. The search included peer-reviewed original research articles with no date restriction (from inception to August 12th, 2024). The final search strategy was reviewed and validated by a research librarian to ensure methodological rigor. The complete search strategy is available in Table 1.

**Table 1:** Search strategy for WLST decisions for patients with a severe ABI. All terms were included in the search. Each term within a column was separated by the Boolean operator “OR”, and each column was combined using “AND”.

Concept 1	Concept 2	Concept 3	
ABI (population)	Withdrawal (decision)	Life sustaining therapy (intervention)	
“brain injur*” “neurological injur* “Cerebrovascular disorder*” “stroke*” “brain infarction*” “intracranial hemorrhage* “brain aneurysm*” “intracranial aneurysm*” “subdural hematoma*” “diffuse axonal injur*” “disorder* of consciousness” “consciousness disorder*” “altered state* of consciousness” “minimally conscious state*” “unresponsive wakefulness syndrome*” “Vegetative” “coma”	“withdraw*” “Cessation” “interrupt*” “terminat*” “remov*” “Ending”	“life support” “medical support” “life sustaining*” “end of life care” “Terminal Care” “critical care” “Palliative Care” “mechanical ventilation” “artificial respiration”	Title/Abstract
“Brain Injuries” “trauma, nervous system”[MeSH Terms:noexp] “Cerebrovascular Trauma” “Craniocerebral Trauma” “Cerebrovascular Disorders” “Stroke” “hypoxia, brain” “Intracranial Hemorrhages” “Intracranial Aneurysm” “Consciousness Disorders”	“Clinical Decision-Making” “Advance Care Planning”	“Life Support Care” [MeSH Terms:noexp] “Resuscitation Orders” “Terminal Care” “Palliative Care”	MeSH Term

All terms were included in the search. Each term within a column was separated by the Boolean operator “OR,” and each column was combined using “AND”.

### 2.2 Eligibility criteria

Inclusion criteria included:

1. Original peer-reviewed studies.
2. Involving adult patients (≥18 years) with severe acquired brain injury, including (but not limited to) traumatic brain injury (TBI), subarachnoid hemorrhage (SAH), intracerebral hemorrhage (ICH), ischemic stroke or anoxic brain injury.
3. Explicitly discussing WLST decision-making, including influencing factors, processes, or outcomes.

Exclusion criteria included literature reviews, editorials, commentaries, notes, articles in languages other than English or French, pediatric and non-stratified results across diagnosis types. Guidelines were also excluded to more accurately reflect real-world clinical decision making. The studies combining patients with different critical conditions such as cardiac arrest, sepsis, and severe acquired brain injury into one group without analyzing data for ABI were excluded. Brain-dead patients, as well as those in a conscious state capable of participating in WLST decision-making, were also excluded from the study.

### 2.3 Data extraction

Article selection was carried out independently by two reviewers (AA and RJ), who initially screened titles and abstracts for relevance, followed by full-text assessment of potentially eligible studies. Any discrepancies during the selection process were resolved through discussion to reach consensus. A subset of studies with persistent disagreement was reviewed by a third investigator (CD) for final inclusion or exclusion, ensuring methodological consistency and reducing selection bias. Reference lists of included studies were also screened to identify any additional eligible sources.

Following study selection, data were systematically extracted by two independent reviewers (AA and CR) using a standardized spreadsheet template. The extracted variables included study characteristics, population details, methodology, and factors influencing WLST, among others, in accordance with the predefined review objectives.

## 3 Results

### 3.1 Findings

**Figure 1:**
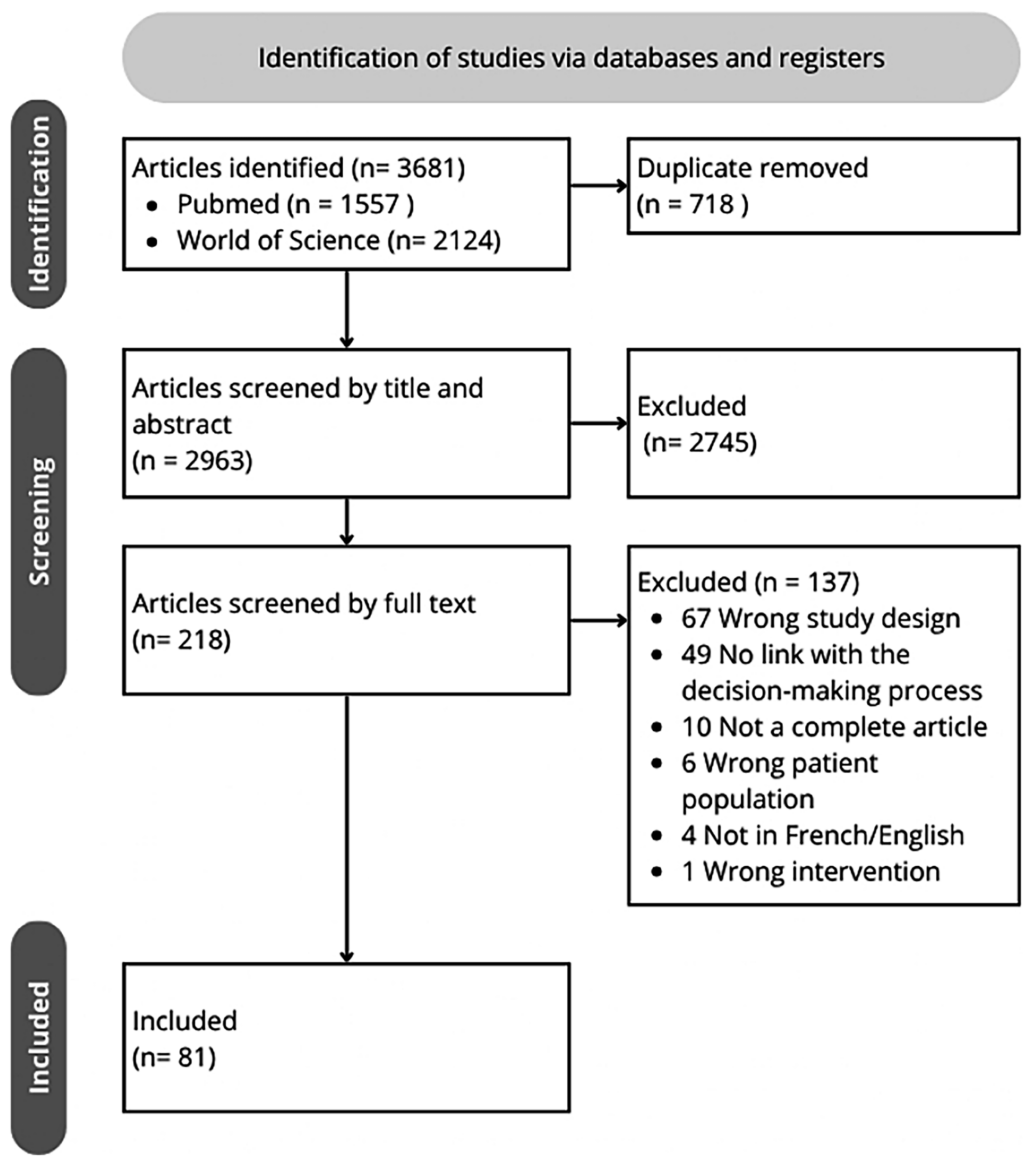
PRISMA-ScR flow chart.

In total, *N* = 3,681 studies were found using the keywords in the two search bases used. After rejecting duplicates, 2,963 articles were screened by title and abstract, of which 218 passed to the full text screening phase. After excluding 136 papers that did not fit the inclusion criteria, 81 articles were included in this review (Figure 1). The characteristics and findings of each included article was extracted.

**Figure 2:**
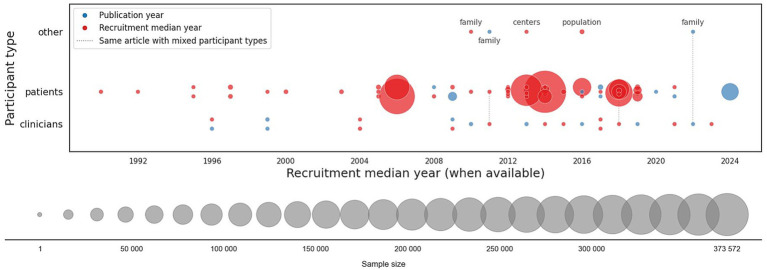
Distribution of included studies according to recruitment period (when available) and sample size. Bubble size reflects sample size (see size legend); colors indicate whether the displayed date corresponds to the recruitment period or the publication date; dotted lines link multiple entries from the same article across participant groups.

81 included articles ranged from 1995 to 2024. Publication activity peaked in 2022 and 2024, with 8 and 10 included articles respectively, representing 22% of the total, the equivalent of all articles between 1995 and 2011. Figure 2 presents the included studies according to recruitment period (when available) and sample size. Of the 59 articles directly including patients, 23 (39%) focused on TBI, 20 (34%) on stroke (one or multiple types), 5 (9%) on anoxic brain injury, and 11 (19%) on diverse brain injury types. Studies principally originated from the United States (44/81, 54.3%). The others were from Australia (*n* = 2), Belgium (*n* = 1), Canada (*n* = 7), Japan (*n* = 4), United Kingdom (*n* = 2), Netherlands (*n* = 3), Switzerland (*n* = 1), France (*n* = 3), Ireland (*n* = 1), Germany (*n* = 2), Denmark (*n* = 1), Brazil (*n* = 1), Norway (*n* = 1), or were conducted internationally in various groups of countries (*n* = 6). Included articles presented various methodologies, the most frequent being the retrospective quantitative medical chart analysis (44/81), with a few qualitative prospective studies with interviews (10/81) or survey designs (14/81). One study used an ethnographic methodology, 5 were prospective observational studies, 4 were case studies and one combined survey and prospective observational data.

Analysis of the contents revealed eight categories of factors that were documented to shape WLST decisions in severe ABI patients: demographic factors (*n* = 50), prognosis and clinical factors (*n* = 59), family preferences (*n* = 28), physician-related factors and institutional context (*n* = 31), formal medical directive (*n* = 13), ethical/legal frameworks (*n* = 13), geographical differences (*n* = 9) and religious beliefs (*n* = 5).

### 3.2 Demographic factors

#### 3.2.1 Age

Out of the 81 extracted articles, 49 studied age as a factor influencing occurrence and/or timing of WLST: the older the patient, the higher the odds of undergoing WLST. Indeed, each additional year of age is associated with a significantly increased risk of typically 2–6% (8–12). The same broad conclusion emerged from Bhogadi et al., which reported a 35% increased risk per 5 years of age (13), as well as Grossestreuer et al. and Rubin et al., which noted, respectively, a 18% and a 34% increased risk with each decade (14, 15). Becker et al. found an even higher increment in risk of WLST with age (OR ~1.6 per decade), as well as a decreased likelihood of receiving surgical intervention (OR 1.82 per decade) in ICH patients (16), the latter finding being corroborated by Tran et al. (17). Furthermore, DeMario et al. compared WLST decisions across age groups using 19-year-olds as the reference point and found that the odds of WLST increased progressively with age—from a 26% increase in the 20–29 age group to over a 2000% increase in individuals over 90 years old (18). Interestingly, Pokryza et al. denoted that despite the increasing odds of WLST with age, patients aged over 90 years old were more likely to be discharged than to receive WLST (19). Finally, stroke patients older than 70 have a significantly higher and earlier risk of experiencing WLST with more than twice the hazard compared to younger patients, based on results from de Montmollin et al. (20).

Multiple studies explored the influence of age by comparing patients who received WLST to those who did not (21–31). For instance, Kowalski et al. (30) and Ge et al. (31) reported higher mean and median ages in the WLST group compared to non-WLST patients. Interestingly, Tran et al. showed that this effect is also seen when only recruiting young patients: WLST patients were significantly older than those who did not undergo WLST (29 vs. 27, *p* < 0.001) (26). Furthermore, older age was also found to be associated with a shorter delay to WLST, as seen in Tran et al., DeMario et al., and Kwak et al. (17, 18, 32). The latter study also showed that age more strongly predicted early WLST than injury severity at admission and thus may have the greatest influence in the decision-making process. A recent paper by Bath et al. similarly found that age was a more determining factor in timing of WLST than prognosis: compared to geriatric patients (> 65), adult patients (18–64) met more of the poor prognosis indicators but still had a longer delay in WLST (33). A few more quantitative studies showcased the influence of age on WLST occurrence and timing (34–47).

In survey and interview methodologies, age was observed as influencing decisions for both the clinicians (48–52) and the general population, when asked to play the role of a hypothetical surrogate decision maker (53). Furthermore, moral and ethical challenges related to WLST decisions often vary depending on the patient’s age group. Younger patients were more commonly associated with the pursuit of invasive and curative measures. A discourse analysis study by van der Riet et al. (54) described how patient age could evoke emotional tension among healthcare providers when questioned in interviews.

#### 3.2.2 Sex and gender

Sex and gender characteristics were often mentioned in studies, but the analysis of its influence on WLST was scarce (only 20/82 studies). When sex was included in analysis, almost half of the studies (8/20) did not find a significant association between patient sex and the likelihood of WLST (8, 16, 29–31, 40, 43, 46). In the remaining studies, results were contradictory. In a large retrospective analysis on SAH patients, Qureshi et al. (21) found that women were slightly more likely to receive WLST than men (OR 1.2, 95% CI 1.0–1.3) as was found in 6 other studies (10, 14, 23, 25, 32, 44). Conversely, a large database study analyzing predictors of WLST in TBI patients found that women were less likely to undergo WLST (OR 0.91, 95% CI 0.84–0.98) and experienced a longer median time to WLST compared to men (3 vs. 2 days) (18). Those same conclusions were drawn in 4 other studies (9, 13, 19, 53).

#### 3.2.3 Race and ethnicity

Ethnicity and race have been explored as potential factors influencing end-of-life decision-making: 25 out of the 82 included articles conducted analysis on that matter. While Kowalski et al. (30) and Zurasky et al. (47) did not find any significant correlation between patients’ race and WLST, most articles noted that White patients underwent WLST more frequently (9, 12, 13, 15, 18, 19, 21, 25, 26, 28, 34, 36, 37, 39–41, 44, 46, 55, 56), and earlier (32, 57), than any other race. More specifically, non-Hispanic Black and Hispanic patients were consistently the least likely to WLST, with a 30% (OR 0.7, 95% CI 0.5–0.8) and 60% (OR 0.4, 95% CI 0.3–0.6) reduced likelihood, respectively (21). Further analysis on race is sparse: 23 out of the 25 studies that included race were conducted in the US, and the included races were often limited to White/non-White (13, 15, 28), White/non-White/Hispanic (34) White/Black/others (19, 36, 46) or White/Black/Hispanic/others (21, 26, 30, 37, 44), with Asian patients being underrepresented. However, the only international study that included race, a study from Muñoz Venturelli et al. noted a significantly lower rate of WLST in patients of Chinese ethnicity compared to non-Chinese, without specifying the race (8). Interestingly, regarding treatment aggressiveness at the end of life, do-not-resuscitate (DNR) orders were more frequently issued for White patients (117; 18%) compared to Black (27; 12%) and Hispanic (49; 13%) patients (*p* = 0.03) (55).

#### 3.2.4 Socioeconomic status and insurance

Socioeconomic status (SES) and health insurance coverage were addressed in several articles (*n* = 11); however, their overall influence on WLST decisions appeared inconsistent and difficult to interpret due to the presence of confounding factors. Furthermore, the fact that all included studies originated from the United States limits the geographical representativeness of the findings. Fraser et al. found that patients with higher estimated income were more likely to only receive comfort care instead of curative care (36). Additionally, Melmed et al. showed that patients residing in wealthier neighborhoods (median household income: $88,687 vs. $77,350, *p* < 0,001) were more likely to have WLST, even after adjusting for age and brain injury severity (41). In a scenario-based survey, Garg et al. further showed that an annual income of less than 25 K$ was predictive of choosing life sustaining measure for an hypothetical relative in vegetative state (53).

Insurance status findings presented a paradoxical pattern relative to income effects. Tran et al. (26) found that having health insurance was associated with a decreased likelihood of undergoing WLST, while Malhotra et al. (39) and Melmed et al. (41) further reported that privately insured patients were even less likely to undergo WLST compared to those with public insurance. These results can suggest the influence of healthcare financing on treatment continuation. However, when considering self-paying (uninsured) patients as well, the pattern is not linear: the likelihood of WLST does not increase in a straightforward gradient from private to public to no insurance. Indeed, a large (*n* = 37,931) multicenter study by Williamson et al. (46), noted that compared to patients with private insurance, those with Medicare were 55% more likely to undergo WLST, while self-pay patients were only 36% more likely. The same conclusions were drawn by Bhogadi et al. (13). Alkhrachoum’s findings align with these results as well: patients with WLST were more likely to have Medicare insurance (53% vs. 44%), but less likely to be uninsured (8% vs. 13%) (25). This non-linear relationship suggests that the influence of insurance status on WLST decisions is complex and likely involves multiple interacting factors beyond insurance type alone. Notably, this complexity is further illustrated by the apparent inverse effects of income and insurance status on WLST rates, despite higher income typically correlating with better insurance coverage.

In keeping with this idea, Qureshi et al. (44) concluded that having Medicare/Medicaid insurance was a predictor of WLST, while Hornor et al. (57) showed WLST tends to occur later in privately insured patients. These results further suggest that insurance status may influence not only the likelihood but also the timing of end-of-life decisions.

#### 3.2.5 Others demographical factors

Melmed et al. (41) revealed that multiple sociodemographic factors, including being married with a low income and unemployment, were associated with lower rates of WLST in ICH patients. Similarly, Kwak et al. (32) reported that lower education levels (less than high-school diploma) were associated with a higher likelihood of early WLST. Garg et al. also explored the influence of spoken language in a US-based, English-dominant healthcare context and showed that having English as a non-native language was associated with lower rates of WLST (53). On the contrary, Kwak et al. also conducted in the United States, found that limited English proficiency was associated with higher proportions of early limitation of LST (58).

### 3.3 Prognostic and clinical indicators

59 articles reviewed mention prognosis and clinical characteristics as a significant factor for WLST in patients with severe ABI’s (8–14, 16, 17, 19–22, 24–26, 28–32, 34–40, 42–48, 50–52, 54, 56, 57, 59–74). Muñoz Venturelli et al. (8), found WLST was carried out in patients with established predictors of poor prognosis, who were consequently more likely to experience death or survive with severe disability. Similarly, in a multicenter cross-sectional study by Van Veen et al. (49), 60% of centers reported that ≤50% of ICU deaths in severe neurological injury were preceded by WLST, which tended to be delayed in TBI patients with poor prognosis. De Angelis et al. (10) reported that in 83% of patients with limitations of care, final decisions were made within 14 days, with higher injury severity associated with earlier implementation. In contrast, Côte et al. (35) found no association between Glasgow Coma Scale (GCS) scores and WLST decisions in traumatic brain injury patients.

Prognosis following an ABI is often less predictable than in other medical conditions, such as advanced cancer, and may vary depending on the specialty and experience of the physician responsible for prognostication. In a discourse analysis by van der Riet et al. (54), the predictability of a patient’s prognosis strongly influenced decision-making around WLST, specifically medically assisted nutrition and hydration.

Clinical factors such as Glasgow Coma Scale scores, pupil reactivity, and overall injury severity were consistently used to guide prognostication. In a study by Malhotra et al. (67), greater injury severity based on intracranial and extracranial trauma, midline shift, and pupil asymmetry was associated with a higher likelihood of WLST. In a cross-sectional study by Turgeon et al. involving intensivists, neurosurgeons, and neurologists, most physicians (65%) considered accurate prognostication most valuable within the first week of ABI, relying primarily on bedside monitoring, clinical examination, and imaging, while fewer utilized electrophysiology or biomarkers (71). In a multicenter cross-sectional study, Bozkurt et al. (50) reported that additional clinical factors, particularly comorbidities such as end-stage organ failure (68.3%), malignancy (51.9%), ischemic cerebrovascular disease (35.2%), congestive heart failure (31.4%), and dementia (22.3%) also influenced neurosurgeons’ decisions regarding WLST. Supporting this, a retrospective cohort study by Van Erp et al. (29) reported that patients in the WLST group had a greater burden of pre-existing comorbidities, evidenced by higher ASA physical status classifications (*p* = 0.002). Similarly, Bhogadi et al. showed that WLST rates were significatively higher in frail patients than in non-frail patients (13). Furthermore, in a simulated decision-making study, Albrechtsen et al. (75) found that although magnetic resonance imaging (MRI) revealed additional or more extensive pathology in 81% of patients, it did not consistently correlate with changes in clinical decisions.

While prognosis often refers to survival, it can broadly include expectations about the patient’s future functional status, comfort, and quality of life. For instance, in a study by Muñoz Venturelli et al. (8), the authors found that WLST decisions were often shaped by a prognosis of severe disability rather than death. Similarly, in a retrospective cohort study of terminally extubated ICU patients, WLST decisions were largely influenced by perceived poor neurological prognosis and expectations of limited quality of life, with less emphasis placed on prolonging life. Consistent with these findings, O’Callahan et al. (43) reported that WLST decisions were strongly tied to predicted poor functional outcomes, underscoring how the perceived futility of sustaining life with profound disability frequently directed decision-making.

### 3.4 Family preferences

Across 28 articles, the literature explores the complex and often burdensome role of family members or legal representatives in making decisions regarding WLST for patients with severe ABI who lack decision-making capacity.

In the study by Bozkurt et al. (50), neurosurgeons reported that family members were “very” or “somewhat” involved in the decision-making process for over 80% of TBI patients. Similarly, O’Callahan et al. (43), found that family members were available to participate in 96% of the 24 cases in which life support was withheld or withdrawn. Additionally, in an ethnographic study by Frey et al. (76), the authors highlighted that in the event where ABI patients are incapacitated, much of the decision-making authority rests with the family, representing a primary source for reconstructing the patient’s past wishes. This surrogate-representation role is pivotal, as illustrated in a hypothetical-case survey by Asai et al.: only 3% of Japanese physicians would withdraw artificial alimentation and hydration when a patient had no family to reconstruct the patient’s wishes, whereas 17% would do so when both prior wishes and family agreement were present (59). The central role of families in the decision-making process was also highlighted in multiple other papers (29, 30, 34, 38, 40, 46, 49, 52, 53, 62, 66, 68, 73, 77–83).

In a retrospective observational study by Wakatake et al. (73), the authors observed a discrepancy between the number of families requesting WLST and the actual implementation, with only 19 WLST out of 40 requests. Family requests were not always fulfilled, with decisions delayed or declined due to legal constraints, differing medical opinions, or institutional policies.

In a qualitative exploratory study, van der Riet et al. (54) described how family preferences may shape WLST through requests for continued nutrition or hydration, often driven by fears of starvation, the symbolic value of feeding, or the wish to prolong life for meaningful events. In the context of poor neurologic prognosis, Ong et al. (48) found that clinicians may strengthen their prognostic opinions to align with what they believe families expect, indicating that perceived family preferences can directly influence how prognosis is communicated and, in turn, shape WLST decisions. Additionally, in a retrospective observational study by Diringer et al. (9), when families opposed withdrawal of mechanical ventilation, life support was continued despite an unfavorable prognosis.

### 3.5 Physician related factors and institutional context

Across 31 articles, WLST approaches varied according to physicians’ belief systems, specialty, level of experience, as well as the type and location of healthcare institutions. The primary decision-makers in WLST were the attending physicians (84) and the patients’ surrogate decision-makers, with significant differences in how end-of-life care was approached. A recurring theme in the literature was the variability in physicians’ prognostication practices and definitions of futility, and how this variability may influence decisions regarding WLST (59, 70, 71, 77, 83). For instance, in a case-based survey study by Rogge et al. (69) prognostic expectations and WLST considerations varied according to physician experience, with those with sufficient exposition in rehabilitation medicine more often anticipating favorable outcomes and opting to continue treatment compared to colleagues without such experience. Ge et al. (31) highlighted marked inconsistencies among clinicians in their prognostic assessments, an important finding given that, as shown in a prospective quantitative study by O’Callahan et al. (43), physicians most often base WLST decisions on a perceived poor prognosis. However, as demonstrated in a retrospective cohort study by Hornor et al. (57), at times clinicians are unable to accurately predict the survival of a seriously injured patient as well as their long-term functional or cognitive outcomes.

Interestingly, Mebane et al. specifically studied the influence of physician’s race, age, and gender on EOL attitudes. In their survey, more White physicians than Black physicians considered tube feeding in terminally ill patients “heroic” (58% vs. 28%, *p* < 0.001) and viewed physician-assisted suicide as acceptable (36.6% vs. 26.5%, *p* < 0.05) (85).

Physician education experience has also been identified as a factor influencing WLST decisions. In a cross-sectional survey-based study by Forte et al. (86), physicians with previous end-of-life (EOL) education and greater familiarity with EOL issues were more likely to implement written DNR orders, involve nurses in decision-making also in Kerever et al. (79), and engage in discussions about withdrawal, whereas those with less exposure to EOL training or literature tended to favor full-code approaches, delay withdrawal decisions, or act more cautiously due to legal concerns. Contrarily, in a case-based survey study by Rogge et al. (69), surveying neurologists, the authors found that the decision for WLST was not influenced by gender, age, or length of clinical experience. However, the intervention chosen was in fact associated with prior experience in specialized neurologic rehabilitation settings.

On the other hand, in a cross-sectional multicenter descriptive study by Bozkurt et al. (50), the neurosurgeon’s age was positively associated with WLST decisions. Interestingly, language seemed to play a role in comfort regarding WLST decisions. In a cross-sectional survey by Dierickx et al. (78), Belgian physicians showed notable linguistic differences, with Dutch-speaking clinicians more often considering withdrawal of artificial nutrition and hydration appropriate (63%) compared to French-speaking clinicians (39%).

Objectives of care vary by specialty. In a survey comparing neurosurgeons and ICU physicians, both valued quality of life in WLST decisions, but neurosurgeons ranked it as the primary factor, whereas ICU physicians prioritized avoiding a persistent vegetative state (87). Interestingly, in a study by Becker et al. (16), the authors suggested that neurosurgeons generally adopt a more aggressive approach to patients with ICH, opting for surgical intervention both earlier and more frequently than neurologists. In a single-center qualitative study by Ong et al. (48), neuro-intensive care unit (NICU) intensivists tended to base most decisions on official guidelines, whereas non-NICU clinicians more often relied on personal experience and specialty-specific knowledge to guide their practice. In Collins et al., intensive care physician were two times more likely to initiate EOL discussion than any other physician (62). Lastly, physicians in different specialties may also interpret neuroprognostic tools differently leading to differences in prognostication. In a retrospective single-center cohort study by Beekman et al. (60), the inter-rater reliability of early head computed tomography for detecting hypoxic–ischemic brain injury between radiology and three neurointensivists varied widely, with Kappa–Fleiss test values ranging from 0.13 to 0.66.

Institutional factors such as geographic location and university affiliation have been shown to influence the course of WLST. Malhotra et al. and Pokrzywa et al. (19, 39), showed that patients treated at nonprofit centers had higher odds of undergoing WLST compared to those treated at for-profit centers. Similarly, Qureshi et al. (44) reported that in patients with post-thrombolytic ICH, teaching hospitals (*p* = 0.01) and large hospitals (*p* = 0.05) were more likely to withdraw care. Related findings were noted in Alkhachroum et al. and Williamson et al. (in severe TBI) (25, 46). In a prospective cohort study, Cooper et al. (34) further found that withdrawal of care orders were more common in high level trauma centers and in intensivist-led ICU, findings that were corroborated by Bhogadi et al., Gambhir et al., and DeMario et al. (13, 18, 37). Finally, Manara et al. discussed the availability of resources (e.g., ICU beds) as a potential factor influencing WLST decisions (68).

### 3.6 Formal medical directive

13 articles identified formal medical directives (including DNR orders) as contributing factors in WLST decision-making, where the medical directive was either established by the patient themselves before the brain injury or by the surrogate decision maker at time of ICU stay (10, 11, 13, 16, 23, 26, 29, 59, 61, 65, 80, 82, 88). In a retrospective observational study by Shaw et al. (12), the presence of a pre-existing DNR was positively associated with withdrawal of life support (OR 3.22, 95% CI 1.03–10.50). Similarly, van Erp et al. (29) reported that the high proportion of early treatment-limiting decisions in their cohort may be partly attributable to the presence of DNR orders established by the surrogate. Furthermore, De Angelis et al. (10) found that 14% of patients who died following a limitation of care had a DNR; however, most patients with an initial DNR subsequently progressed to more restrictive measures, including no escalation of care or WLST, prior to death.

A case report also illustrated the role of DNR orders in an ethically and medically complex context. Krieger et al. (80) described a 63-year-old man with a history of suicidality who sustained an ICH following a suicide attempt. He remained awake but non-communicative and ventilator-dependent, and with no improvement in mental status, his estranged brother signed a DNR to honor the patient’s previously expressed wishes to avoid prolonged dependency. The hospital ethics committee then convened to consider WLST for this patient.

While rare considering the sudden aspect of brain injuries, some articles mentioned advanced directives established by the patient themselves before the injury as a contributing factor for WLST (13, 16, 23, 26, 65). From a physician perspective, Asai et al. 1999 (59) found, in Japan, that 91% were influenced by written advance directives declining life-sustaining treatment, which they considered significantly more persuasive than oral directives (91% vs. 73%, *p* < 0.0001). Furthermore, in Rogge et al., when a written advance directive supported WLST—if the prognosis for regaining consciousness and communicative abilities was poor”—but the patient’s spouse expressed uncertainty, inconsistencies in physicians’ responses were observed. Specifically, 4% of physicians interpreted the advance directive as indicating a preference for life-sustaining treatment while nonetheless deciding to proceed with WLST, whereas 7% interpreted the patient as not wanting life-sustaining treatment yet chose to provide it (69).

### 3.7 Ethical and legal considerations

Legal frameworks surrounding WLST were discussed in four studies, highlighting substantial variability in how laws influence end-of-life decisions and govern the medical practice of physicians. A case report by Sequeira and Lewis (88) describes a patient without a surrogate decision-maker to illustrate how WLST practices differ under varying US state laws. In New York, the Family Health Care Decisions Act legally prohibits WLST and requires court approval unless treatment is medically inappropriate or death is imminent, often leading to prolonged medical interventions even in cases of permanent vegetative state. In other states, statutory authority for healthcare decisions may rest with the attending physician, while some jurisdictions require the involvement of a second physician, an ethics committee, or, in certain cases, a clergy member.

In a questionnaire-based survey, Swanson and McCrary (89) found that physicians in Texas who demonstrated high legal defensiveness, often due to concerns about malpractice or criminal liability as well as limited knowledge of medical legislation, were more likely to consider interventions “not futile” for patients in a persistent vegetative state. This could lead to continued life-sustaining care and underscores how medicolegal concerns can conflict with ethical duties to avoid futile interventions. Similarly, in a cross-sectional survey-based study by Forte et al. (86), legal concerns, followed by team and societal opinion, were the most common obstacles to following the course of action believed to be best. Legal obstacles were also highlighted in the cross-sectional vignette-based survey of ICU physicians and neurosurgeons by Aita et al., in which concerns about legal repercussions and media scrutiny influenced physician behavior, at times resulting in WLST being carried out discreetly (77).

Ethical considerations were identified in 10 studies as influential factors in WLST decision-making (38, 54, 59, 68, 76, 78, 80, 82, 84, 88). Van der Riet et al. (54) highlighted, through focus groups, the internal conflicts clinicians face when navigating end-of-life care. The central themes were the ‘blurring of boundaries’ between doctors and nurses’ responsibilities and the balance between cure and care, illustrating ethical complexity in the face of prognostic uncertainty. To reduce this uncertainty and discomfort, Manara et al. (68), advises to delay WLST to improve diagnostic clarity and reduce the risk of premature withdrawal or self-fulfilling prophecies. In an ethnographic study by Frey et al. (76), the authors highlighted the ethical dilemmas inherent in tube feeding decisions after severe stroke, where patient incapacity required surrogates and clinicians to infer wishes. These decisions raised questions about respecting autonomy, balancing beneficence and non-maleficence, and managing the moral distress that arose when actions conflicted with perceived patient values.

### 3.8 Geographic and regional differences

Variations in WLST occurrence and/or timing were reported across regions and geographic areas in 9 articles, however were usually not the primary focus of the studies. In an international survey study by Ball et al. (51) almost all of the 419 physicians questioned believed there are regional/geographical variations in WLST. However, only 14–38% reported having formal medical futility laws or government-issued guidelines to guide their practice.

In a retrospective study by DeMario et al. (18), the Midwest and Northeast regions of the United States exhibited the highest odds of WLST, suggesting that regional culture may play a role in shaping these decisions. Alternatively, in a retrospective analysis by Qureshi et al. (44), the study observed that hospitals in the West of the United States [OR 1.7, 95% CI 1.2–2.4] were more likely to implement withdrawal of care. Additionally to the two previously mentioned articles, in a retrospective observational study by Qureshi et al. (21), hospital location in the southern United States (OR 0.7, 95% CI 0.5–0.8) was a significant predictor of a lower likelihood of “withdrawal of care” among patients with SAH. At a smaller scale, some variations in rate of WLST was observed within regions of Florida in Alkhachroum et al. (25). In an international study, Muñoz-Venturelli et al. showed that Chile (28%) had the highest rate of WLST, followed by Italy (13%), the UK (12%) and Australia (10%) (8).

In terms of timing of WLST, in a retrospective cohort study by Hornor et al. (57) the Northeast region (OR 1.18; 95% CI, 1.00–1.39) emerged as the only institutional factor significantly associated with delayed WLST among the regions defined by the US Census Bureau (Northeast, South, West, and Midwest). However, in two studies by van Veen et al. (49, 74), timing did not vary between regions, but the occurrence of WLST did, ranging from 0% in Eastern Europe to 96% in Northern Europe.

### 3.9 Religious factors

Religious beliefs have been found to be associated with the decision making of both surrogate decision makers and physicians in 5 articles. Indeed, in a scenario-based survey by Garg et al. (53), the hypothetical vegetative patients received more life-sustaining treatments when the respondent, playing the surrogate decision-maker, self-identified as Evangelical Protestantist (OR 3.72, 95% CI 1.28–10.84) or Catholic (OR 4.01, 95% CI 1.72–9.36), comparatively to individuals not affiliated with religion. Furthermore, while hard to objectively quantify, the influence of religious beliefs (or lack thereof) of physicians on WLST can be studied in self-reported surveys, such the one by Ball et al. (51). In this international study, 71% of clinicians from Asia reported that they believe their faith had an impact on the end-of-life decisions made in their practice. For North America and Europe, only 6–24% of physicians reported this belief, although that statistics could be skewed by the fact that Canadian and European respondents were mostly atheist or agnostics (51). Forte et al. also reported the surveyed physician’s religiosity, but no conclusion was drawn on its influence on WLST decisions (86).

Furthermore, the patient’s religion is also correlated to WLST occurrence. In a retrospective cross-sectional study by Melmed et al. (41), compared to non-religious patients, christian patients experienced higher rates of WLST (66% vs. 56%, *p* < 0.05). This same study denoted that individuals identifying as non-religious or atheist were less likely to undergo WLST and had significantly lower mortality (*p* < 0.05 for both outcomes).

Only one article reported an adaptation of the decision-making process based on the patient’s religion: Pan et al. (82) described a case involving an Orthodox Jewish family where religious authorities (a Rabbi) had to be consulted before mechanical ventilation could be withdrawn.

## 4 Discussion

This scoping review set out to identify the diverse factors that influence decisions to withdraw life-sustaining treatments (WLST) in adult patients with severe acquired brain injury (ABI), and map the breadth, depth, and nature of evidence supporting the influence of these factors on the decision-making process. We synthesized 81 studies published between 1995 and 2024, which showed that, despite wide heterogeneity in methodologies and contexts, several consistent themes emerged, reflecting the complexity of end-of-life decision-making in neurologically devastated patients. Decisions surrounding WLST appear to arise from an interplay between clinical prognostication, patient characteristics, surrogate and physician beliefs, institutional norms, and broader sociolegal and cultural environments.

### 4.1 Multifactorial nature of WLST decision-making

Across studies, WLST decisions were seldom based on a single determinant. Instead, they reflected a convergence of medical and non-medical factors that collectively shaped perceptions of prognosis and futility. Demographic and clinical variables, especially age, injury severity, and comorbidities, were the most frequently cited influences, often correlating with a higher likelihood and earlier timing of WLST. However, their weight appeared to exceed what would be justified by objective clinical criteria alone.

The recurrent finding that older age predicts WLST more strongly than injury severity underscores a potential normative bias in how age is equated with quality of life or recovery potential. It may also confirm the well-documented systemic ageism reported in the healthcare system (90). While older patients are less likely to desire aggressive treatment (91), this wish is overgeneralized. This misconception of patients’ care preferences may further influence the occurrence of WLST in the brain-injured population, as their inability to communicate wishes makes them particularly vulnerable. The increase of WLST in older age could also be due to the tendency of surrogate decision-makers to under evaluate quality of life (92, 93), leading them to believe that the projected quality of life of their loved one is not acceptable. Furthermore, age may act as a confounding factor for several other determinants of outcome, including comorbidities, premorbid functional status, and frailty. As a result, the apparent effect of age per se may be overstated, as it is partly mediated by its strong association with these established poor-prognostic indicators rather than reflecting an independent influence of chronological age alone (94, 95).

Sex and gender were often briefly mentioned but rarely analyzed in depth regarding their influence on WLST. Among studies that included sex, nearly half found no significant association, while others reported conflicting results: some found women more likely to undergo WLST, whereas others found women less likely and experiencing longer times to WLST compared to men. This inconsistency could be explained by the unequal distribution of sex in some etiologies: for example, men are almost twice as likely to suffer a TBI than women in the US (96, 97). Such differences in sampling between groups could significantly impact statistical outcomes and observed associations with WLST. However, multiple studies show that women tend to prefer less aggressive treatments compared to men in cardiology cases (98), which could explain why some included studies found them to undergo more WLST. Importantly, because of the inability of patients to communicate, and therefore to self-declare their gender, the analyses are based on attributed sex exclusively. Additionally, the absence of sex- and gender-sensitive analyses in survey- and interview-based studies, prevented studying how it may influence WLST. For example, in a study by Ong et al., 30 physicians were surveyed, 27 male (90%) and 3 female (10%), yet they did not discuss sex as a contributing factor in WLST decision-making for clinicians (48).

The literature seems to suggest that White patients are the most likely to WLST - and earlier - while non-Hispanic Black patients and Hispanic patients are the least likely. This difference between races could be explained by a few mediating factors. Multiple studies have documented that African Americans tend to prefer more aggressive treatments in end-of-life and thus could be the results of respected wishes (99, 100). Second, racial minorities in the US are usually more religious than non-Hispanic White (101), which can influence perspectives on medical interventionism. For example, in a study by Johnson et al. (99), African Americans were less comfortable discussing death and were more likely to have spiritual beliefs in conflict with palliative care. Third, lack of trust in the healthcare system could explain the reluctance of racial minorities to accept poor prognosis and recommendation of WLST (102–104). However, the conclusions that can be drawn from the included articles are limited due to the underrepresentation of some races and ethnicities, including Asian and Indigenous groups.

SES and insurance status was studied as a contributing factor to WLST in 8 studies, all originating from the US. This may be explained by the country’s predominantly private healthcare system, where access to care, treatment decisions, and continuation or withdrawal of life-sustaining therapies can be influenced by insurance coverage, out-of-pocket costs, and socioeconomic disparities. However, studies conducted outside of the US did not specify the structure of their healthcare systems, which limits the ability to draw worldwide conclusions regarding the influence of private versus public healthcare on WLST decisions. Included articles showed that higher the income, higher are the chances of WLST. However, for insurance status, the relationship with WLST is non-linear: Medicare patients show higher WLST rates than both privately insured and self-pay patients, suggesting that factors beyond insurance type influence the decision-making process. This relationship is likely confounded by eligibility criteria for public insurance, which in the United States are largely age-based (105). The absence of studies outside of the US limits the conclusions that can be drawn in regard to insurance status, since the impact of universal healthcare was not quantified. Another mediating factor that can be explored to explain the relationship between SES and WLST decision-making is health literacy. Indeed, some studies showed that lower income was associated with lower health literacy (106, 107). This diminished knowledge and competence to access and take decisions concerning healthcare (108) can influence the discussions with the care provider and the decision-making process. This process can further be impeded by spoken language (either native or non-native), influencing not only the family’s comprehension of complex medical information and the decision-making process, but also how the patient’s values and wishes are expressed and interpreted (109). SES impact on WLST can also be influenced by its relationship with race (110).

Overall, the inconsistent associations between sex, socioeconomic status, and insurance coverage point to the influence of contextual and systemic inequities rather than strictly medical considerations.

### 4.2 Prognostic uncertainty and the risk of the self-fulfilling prophecy

A central tension highlighted across studies concerns the accuracy and timing of neurological prognostication. Physicians frequently acknowledged uncertainty in predicting meaningful recovery, especially in the early phase after ABI, yet WLST often occurred within days of admission. Poor neurologic prognosis consistently emerged as a central driver of WLST decisions in severe ABI (8, 33, 49); however, its influence was often tempered by uncertainty in the first 72 h of care and varied across physicians and institutions (17, 30). Several authors warned that early withdrawal may risk creating self-fulfilling prophecies, where pessimistic expectations limit opportunities for recovery. Early prognostication in severe ABI is difficult and very uncertain, which is the main reason why the Neurocritical Care Society recommends delaying the WLST decisions for at least 72 h (111). Furthermore, there are documented cases where WLST was delayed for organ donation, with patients surviving and recovering well (68). In support, a study by Sadaka et al. reported that, at 6 months post-ABI, 14.5% of patients who initially presented with a GCS score of 3 achieved a favorable outcome (based on the Glasgow Outcome Scale) (112). Overall, the variability observed across specialties and institutions further reflects the absence of unified prognostic frameworks and the limited integration of emerging modalities such as neurophysiology, neuroimaging, and biomarkers in decision-making. Indeed, in a cross-sectional study by Turgeon et al. involving intensivists, neurosurgeons, and neurologists, most physicians (65%) considered accurate prognostication most valuable within the first week of ABI, relying primarily on bedside monitoring, clinical examination, and imaging, while fewer utilized electrophysiology or biomarkers (71). Although not directly addressed in the included literature, growing evidence indicates that some behaviorally unresponsive patients retain preserved brain activity and signs of covert consciousness (e.g., minimally conscious state star (MCS*), cognitive motor dissociation), which may complicate decisions regarding withdrawal of life-sustaining therapy (113). In this context, multimodal neuroimaging and computational modeling approaches hold promise for providing complementary, objective information about brain network function that could inform prognostic assessments and shared decision-making (114). These findings underscore the need for caution, interdisciplinary collaboration, and temporally staged reassessment of prognosis before irreversible decisions are made.

Interestingly, the way the prognosis is communicated to the decision-maker can also influence the final decision. For instance, Ong et al. noted that clinicians often invoked prognosis to reframe expectations, either by emphasizing the likelihood of poor outcomes to encourage alignment with WLST, or by acknowledging prognostic uncertainty as justification to continue treatment (48). Thus, even when families resisted WLST, prognosis remained central by anchoring the terms of discussion between surrogates and clinicians. What is, the way the surrogate-decision makers understand said prognosis also influences the WLST decision process: a cross-sectional survey by Garg et al. (53) demonstrated that the interpretation of prognosis is often shaped by surrogates’ own perspectives, reflecting their values, medical knowledge, religious beliefs, and level of trust in medical workers/systems. This underscores the importance of structured education and training to improve how prognosis is discussed and integrated into WLST decision-making in ABI patients.

Finally, the central dilemma in WLST decisions often lies in whether medical interventions meaningfully improve a patient’s quality of life or simply prolong suffering as prognosis is not always simply about survival. Integrating quality-of-life considerations alongside prognostic indicators is therefore essential to ensure that decisions about WLST align not only with survival metrics but also with the patient’s values and well-being.

### 4.3 Surrogate decision-making and family involvement

Families were almost universally involved in WLST decisions, yet their participation was characterized by emotional burden, decisional conflict, and occasional divergence from clinical recommendations. Studies consistently reported distress among surrogates who felt responsible for the patient’s death, as well as among clinicians attempting to balance respect for autonomy with concerns about futility. Discrepancies between family requests and physician actions—driven by legal constraints, institutional norms, or prognostic disagreement—revealed both ethical complexity and communication challenges. Findings also suggest that clinicians may unconsciously tailor their communication to align with perceived family expectations, which can reinforce pre-existing biases. Structured communication frameworks, ethics consultations, and shared decision-making models may help mitigate these tensions.

Across the 42 studies in which family involvement was described as a central feature of WLST decision-making in ABI, families were reported to be regularly consulted and to play a decisive role in reconstructing the patient’s values and wishes; however, this varied considerably across countries. For instance, in Japan, physicians were more likely to make WLST decisions independently, reflecting a more physician-centered decision model, whereas in North America and Europe, families were systematically included in shared decision-making (12, 71). This discrepancy could be explained culturally: while Western cultures tend to prioritize autonomy (consequently mandating families to represent the patient’s wishes), many Asian cultures value more beneficence, resulting in physician-centered decisions and lower WLST rates (115).

Importantly, the interaction between families and clinicians is bidirectional. While it is expected that families rely on physicians’ recommendations based on their expertise and experience (116), physicians also adapt in function of the families. Indeed, when surveyed, physicians reported tailoring their communication of prognosis in accordance with what they believed the family expected (48). This reciprocal influence meant that family preferences could indirectly shape clinical framing of prognosis, thereby influencing the timing and likelihood of WLST.

Taken together, these findings point to a troubling asymmetry: excessive family influence can prolong treatment felt to be futile, while minimal family involvement increases the risk of physician-driven decisions shaped by implicit biases such as age, cultural background, or socioeconomic status. Across diverse settings, the literature underscores the need for structured communication frameworks that both respect family perspectives and safeguard against undue prolongation or premature cessation of life-sustaining treatment.

### 4.4 Clinician experience, institutional culture, and legal context

WLST practices varied markedly by physicians’ specialty, experience, values, and institutional context. Variability reflected not only individual training and specialty culture but also the medico-legal and educational environments in which care was delivered. Clinicians with prior end-of-life (EOL) education or rehabilitation experience tended to pursue longer observation periods, implement written DNR orders, involve nurses, and engage families in early collaborative dialog (69, 86). In contrast, limited training or fear of litigation often prompted more defensive “full-code” practices. Prognostic framing was likewise shaped by background: rehabilitation-oriented neurologists more often anticipated recovery, whereas intensivists emphasized futility (48, 69). Diagnostic and prognostic uncertainty amplified these differences. Physician demographics also played a role, with older or more experienced practitioners more likely to initiate WLST decisions (50).

At the institutional level, WLST orders were more frequent in trauma centers and closed ICU models than in non-trauma or open units (34). Hospital size, teaching status, and nonprofit designation predicted higher odds of WLST in post-thrombolytic ICH (44), and center-level culture continued to influence practice even after case-mix adjustment (46). Within the US, differences also followed regional and legal boundaries, with higher WLST prevalence in large academic or nonprofit centers and in states with less restrictive statutes (39, 44).

Systemic and structural factors—including ICU resource allocation, bed availability, and broader health-care policies—may further modulate thresholds for WLST (117). Educational gaps reinforce these disparities: less than half of nationally recognized competencies in palliative and EOL care are fully integrated into Canadian medical curricula (118), and US programs show similar inconsistencies (119). Such deficiencies, combined with implicit cognitive biases such as therapeutic nihilism toward older or comorbid patients, likely sustain inter-clinician variability.

Collectively, these findings highlight the need to standardize neuroprognostication (e.g., multimodal tools, defined observation windows), strengthen clinician training in communication and ethical-legal literacy, and embed structured institutional processes—early family meetings, ethics-consult triggers, and time-limited trials—to reduce unwarranted variation and moral distress while aligning practice with patient values (34, 39, 46, 48, 86).

### 4.5 Ethical and cultural dimensions

Ethical reflections within the literature converged on three recurrent dilemmas: defining futility amid uncertainty, balancing autonomy with beneficence, and managing moral distress among caregivers. Religious and cultural beliefs also exerted subtle yet significant influence on WLST practices, with both clinicians’ and families’ worldviews shaping attitudes toward life prolongation and suffering. Cross-cultural variability was evident not only between countries but within multilingual or multiethnic societies. The scarcity of data outside North America and Western Europe limits the generalizability of current findings and highlights the need for culturally sensitive research frameworks that account for pluralistic values and moral reasoning.

WLST decisions were deeply embedded within the ethical and legal contexts of their jurisdictions. Statutory frameworks directly shaped who may authorize WLST and under what conditions, as illustrated in cases of “unbefriended” patients (88). International surveys similarly revealed wide cross-country variability and a general absence of formal futility statutes or government-issued guidance, leaving bedside teams to navigate these decisions without consistent legal support (51). Medico-legal risk perception further influenced practice: in a US physician survey, high legal defensiveness—driven by fear of malpractice and limited health-law knowledge—was linked to a lower likelihood of judging treatment futile in persistent vegetative states, leading to continued life support despite poor prognosis (89).

Ethnographic and discourse analyses described moral distress arising when nutrition, hydration, or ventilation were continued primarily to “do everything,” despite minimal recovery prospects, or when WLST was contemplated under uncertainty (54, 76). Ambiguous definitions of “futility” and “emergency,” coupled with inconsistent professional guidelines, often created tension between overtreatment and undertreatment. Misinterpretation of legal constraints could also foster adversarial dynamics, undermining patient autonomy and delaying WLST even when consensus supported it. The literature therefore emphasizes improving clinicians’ understanding of end-of-life law and expanding the use of group-based decision-making (e.g., ethics committees) to reduce unilateral or legally defensive determinations of futility. Developing clear, locally adapted pathways—such as standardized ethics consultation triggers, time-limited therapy trials, and documentation standards for goals-of-care—could reduce variability and moral distress while aligning decisions with patient values (48, 51, 89).

Geographic and cultural contexts also powerfully shaped WLST practices, reflecting the intersection of social norms, religion, and law. Internationally, physicians from North America, Asia, South Africa, and Australasia acknowledged marked regional variation in WLST (51). Requests for continued artificial hydration and nutrition were common in Southern Europe and Asia, where such interventions hold symbolic and religious meaning beyond medical utility (76). Conversely, Northern European and Canadian cohorts more often withdrew these measures once futility was established, reflecting a stronger emphasis on limiting disproportionate care. Geographic disparities thus illustrate how mortality outcomes after ABI may reflect practice variation as much as biological prognosis. Similar effects are reported in other critical care contexts (120, 121). Harmonized guidelines and culturally sensitive communication frameworks may help mitigate inequities while respecting regional diversity.

Religion influenced WLST at both the surrogate and clinician levels. Protestant and Catholic decision-makers were more likely to request continued life-sustaining treatment, possibly reflecting a belief that divine will should determine death rather than human intervention (100, 122). Conversely, physicians’ self-reported faith had minimal influence on decision-making in Europe and North America but a stronger perceived role among Asian clinicians. These findings are limited by social-desirability bias and uneven geographic representation. Patient religion also played a role, with Christian and Catholic patients more likely to undergo WLST—a pattern opposite to that observed among religious decision-makers—perhaps reflecting differing interpretations of religious values at the bedside.

Taken together, the literature indicates that ethical, legal, and cultural forces do not merely frame but actively shape WLST decisions, influencing both their timing and likelihood. Strengthening clinicians’ ethical and legal literacy, supporting collective deliberation, and fostering transparency about local norms are key steps toward ensuring that end-of-life decisions remain consistent, equitable, and centered on patient values.

### 4.6 Formal medical directives

Formal medical directives, identified in 11 articles, were consistently associated with the implementation of WLST in patients with ABI. The presence of written or oral advance directives, DNR orders, or previously expressed wishes communicated to families facilitated timelier decisions and reduced clinician distress. In Shaw et al., pre-existing DNR orders served as clear indicators of patients’ preferences for less aggressive care, often interpreted by physicians as reliable representations of end-of-life wishes (12). Some patients transitioned from DNR orders to additional treatment limitations, including comfort-focused care or WLST.

Despite their value, such directives remain underutilized. In a meta-analysis of 150 studies (*N* = 795,909), Yadav et al. found that only 36.7% of adults had completed an advance directive and 29.3% a living will, illustrating low prevalence in the United States (123). Limited awareness and misunderstanding were major barriers (124), and the absence of documentation often forced families and clinicians to make critical decisions without explicit guidance, increasing delays and conflicts (12, 23, 26, 40, 82).

These findings underscore the ethical and practical importance of promoting early goals-of-care discussions and systematically integrating advance directives into patient records, particularly in high-risk populations. Encouraging proactive planning could reduce unnecessary interventions, avoid futile care, and better align treatment with patient values.

### 4.7 Strengths and limitations

Our scoping review followed a methodologically rigorous process, including a comprehensive, librarian-validated search strategy and dual independent screening and data extraction. It also integrated studies from diverse disciplines such as medicine, ethics, nursing, and legal studies, offering a multi-layered perspective on WLST that reflects the complexity of real-world decision-making and the range of individuals involved. By including surveys and questionnaires from physicians across specialties engaged in WLST decisions, we were able to capture valuable insights into the internal reflections and thought processes of those directly responsible for making these critical choices.

This scoping review should be interpreted in light of certain limitations pertaining to both the search strategy and existing literature. As for the search strategy, the terms used to describe WLST are likely shaped by sociocultural and linguistic factors. In this regard, our search terms may not have been exhaustive, and publications on this topic that used different terminology to reflect WLST may have been missed. Furthermore, including articles from inception allowed a broader selection of studies, but some may reflect outdated practices, as publication dates span multiple decades. Moreover, the selection criteria allowed the inclusion of studies with mixed populations that were not limited to ABI patients. As a result, some conclusions may reflect broader patient groups and could differ from findings that would have emerged from a strictly ABI population.

The existing literature also presented some limitations to this review. Indeed, most included studies were retrospective or observational, limiting causal inference, and relied on medical records that reflect final decisions without capturing the discussions or contextual factors that shaped them. This limits understanding of the nuanced processes behind WLST. In addition, survey-based studies are subject to self-reporting bias, a limitation that is particularly critical when addressing ethically sensitive issues such as WLST. Furthermore, variability in WLST definitions, thresholds, and reporting further constrained comparability across studies. Importantly, most articles originated from high-income, Western settings—particularly the US—where healthcare systems and legal frameworks differ from those in low-resource or non-Western contexts, limiting global applicability.

Finally, the intersection of race, socioeconomic status, geography, culture, and family preferences was seldom examined holistically, despite their clear interdependence. Collectively, these findings highlight the need for more rigorous and context-sensitive research on WLST in severe brain injury.

## 5 Conclusion

Evidence across the 81 studies indicates that WLST decisions rarely hinge on a single determinant; rather, they emerge from the convergence of patient-level, family-level, clinician−/team-level, and system-level factors. These decisions often unfold in contexts of prognostic uncertainty, emotional distress on behalf of the patient’s family members, and ethical ambiguity, placing a significant burden on both healthcare providers and families. To navigate this uncertainty, it is essential to develop structured, ethically consistent, and culturally sensitive frameworks that can guide clinical practice while respecting patient values and maintaining a realistic approach. WLST decisions are not one-size-fits all. Healthcare providers must embrace the inherent heterogeneity of WLST and opt for a patient-tailored, ethically grounded, and transparent decision-making process that reduces moral distress of clinicians, ultimately promoting more equitable and compassionate care.
